# Association Between Clinician-Level Factors and Patient Outcomes in Virtual and In-Person Outpatient Treatment for Substance Use Disorders: Multilevel Analysis

**DOI:** 10.2196/48701

**Published:** 2023-11-03

**Authors:** Justine W Welsh, Siara I Sitar, Michael J Parks, Samantha C Patton, Jacqueline E Braughton, Lance A Waller, Quyen M Ngo

**Affiliations:** 1 Department of Psychiatry and Behavioral Sciences Emory University School of Medicine Atlanta, GA United States; 2 Butler Center for Research Hazelden Betty Ford Foundation Center City, MN United States; 3 Department of Biostatistics and Bioinformatics Emory University Rollins School of Public Health Atlanta, GA United States

**Keywords:** clinician characteristics, substance use treatment, virtual treatment, in-person treatment, telehealth, patient outcomes, intensive outpatient program, virtual reality, treatment, health care, substance use, data collection, EHR, electronic health record

## Abstract

**Background:**

The use of virtual treatment services increased dramatically during the COVID-19 pandemic. Unfortunately, large-scale research on virtual treatment for substance use disorder (SUD), including factors that may influence outcomes, has not advanced with the rapidly changing landscape.

**Objective:**

This study aims to evaluate the link between clinician-level factors and patient outcomes in populations receiving virtual and in-person intensive outpatient services.

**Methods:**

Data came from patients (n=1410) treated in a virtual intensive outpatient program (VIOP) and an in-person intensive outpatient program (IOP), who were discharged between January 2020 and March 2021 from a national treatment organization. Patient data were nested by treatment providers (n=58) examining associations with no-shows and discharge with staff approval. Empathy, comfort with technology, perceived stress, resistance to change, and demographic covariates were examined at the clinician level.

**Results:**

The VIOP (β=–5.71; *P*=.03) and the personal distress subscale measure (β=–6.31; *P*=.003) were negatively associated with the percentage of no-shows. The VIOP was positively associated with discharges with staff approval (odds ratio [OR] 2.38, 95% CI 1.50-3.76). Clinician scores on perspective taking (β=–9.22; *P*=.02), personal distress (β=–9.44; *P*=.02), and male clinician gender (β=–6.43; *P*=.04) were negatively associated with in-person no-shows. Patient load was positively associated with discharge with staff approval (OR 1.04, 95% CI 1.02-1.06).

**Conclusions:**

Overall, patients in the VIOP had fewer no-shows and a higher rate of successful discharge. Few clinician-level characteristics were significantly associated with patient outcomes. Further research is necessary to understand the relationships among factors such as clinician gender, patient load, personal distress, and patient retention.

## Introduction

### Background

The role of the clinician has been studied as a potential mediator of treatment delivery and patient outcomes in both mental health and substance use disorder (SUD) treatment settings [1-3]. Prior to the forced implementation of virtual services as a result of the COVID-19 pandemic, the influence of clinician-level characteristics on treatment outcomes has been largely evaluated in the context of in-person care, leaving a critical gap to inform the quickly changing treatment landscape of virtual delivery. Historically, virtual services were used more commonly in the treatment of general mental health disorders than for SUDs [4-6]. In March 2020, addiction treatment programs had to rapidly increase the use of telehealth services, often without prior experience or formalized training for their staff in the delivery of virtual treatment. While delivery setting is a critical component of SUD treatment accessibility, retention and outcomes are crucial factors contributing to the quality and effectiveness of these services. This shift created new challenges and opportunities in a novel environment for patients and practitioners alike.

### Role of the Clinician in Treatment Retention and Outcomes

Clinician level of experience such as degree or schooling, training in specific treatment modalities, and time in the field conducting therapy have demonstrated variable results on patient outcomes in in-person settings [1,7-10]. Research examining gender and the racial or ethnic background of clinicians has predominantly tested the potential benefits of matching patients and providers by shared background. Despite clients expressing a preference for a therapist matching their own background or identity, the benefits of matching clients with therapists have been inconsistent [2,11]. Data supporting differences by clinician gender have also demonstrated variability in both the delivery of care and patient outcomes [3,12].

Certain clinician characteristics and specific traits have been implicated in the formation of a therapeutic alliance between patient and provider [13]. Empathy has been recognized as a long-standing important factor in the delivery of quality care [14], an area of focus for clinician training [15,16], and a contributing factor to the formation of a strong therapeutic alliance [17]. For example, robust correlations between the Working Alliance Inventory Bond Scale and the Empathy Scale of the Relationship Inventory (measuring empathy, congruence, and positive regard) have suggested that a vital component of a strong alliance is the therapist’s understanding and relating to patient experience [17]. Therapists with low or distant alliance ratings from their clients may have higher rates of premature treatment disengagement [18], while those with higher facilitative interpersonal skills may also be more effective in changing clients’ symptoms over short periods of treatment (8 sessions or less) [19]. Higher alliance scores have also been associated with greater treatment retention in individuals with Diagnostic and Statistical Manual of Mental Disorders, Fourth Edition (DSM-IV) cocaine dependence [20], an important finding since treatment retention for patients with stimulant use disorder has been found to be lower than other disorders [21]. Additional interactions between alliance and psychiatric severity may also be present, with 1 study finding a strong therapeutic alliance was predictive of treatment completion among patients with opioid use disorder and moderate to severe psychiatric severity compared to those with less psychiatric severity [22].

Initial research suggests that clinician characteristics may interact differently between settings. While therapists might not identify differences when evaluating their own ability to demonstrate empathy and support across in-person versus virtual sessions, patients have described therapists as significantly more supportive and empathetic in remote settings as compared to in-person meetings [23]. To date, there has been limited evaluation of the association between clinician-level characteristics with the use of virtual and in-person treatment platforms and key patient outcomes in SUD-specific treatment programming. The objective of this study is to investigate the potential influence of clinician characteristics on treatment retention and successful discharge through virtual and in-person outpatient services for SUD.

## Methods

### Data Collection

Data were obtained from patients (n=1410) treated at the Hazelden Betty Ford Foundation (HBFF) in their virtual and in-person intensive outpatient program (VIOP and in-person IOP, respectively) [24,25]. This data set has previously been analyzed to investigate the feasibility and effectiveness of VIOP [25], as well as differences in patient demographics and clinical characteristics between in-person and telehealth IOP settings [24]. HBFF is one of the largest national nonprofit providers of addiction treatment services in the United States. As part of program quality and process improvement efforts, HBFF collected prospective data from patients receiving IOP care for substance use–related treatment at HBFF facilities between January 2020 and March 2021. Patients who were discharged from any in-person IOP on or after January 1, 2020, received the IOP-specific outcome surveys in order to capture a comparison group of those who attended IOP only in person. HBFF began piloting VIOP groups in 2019 to better understand the feasibility and acceptability of using a web-based platform for IOP treatment. With the onset of the COVID-19 pandemic, the rollout of the web-based platform was accelerated due to the immediate need for transitioning to in-person treatment. VIOP was developed to be as similar as possible to in-person IOP and included video-based real-time group interactions and individual sessions, leveraging the use of technology that could accommodate low-bandwidth internet connections and ensuring the quality and stability of video feeds during sessions. In-person systems for patient accountability were adapted for virtual care, including crisis or emergency response protocols, privacy monitoring, and random drug and alcohol testing using in-home testing kits or blood alcohol content devices with video support. The VIOP group had just been launched prior to the COVID-19 pandemic but use increased dramatically in response to the greater need for virtual services. Within a 2-week period, 74 IOP groups comprised 541 patients were transitioned from in-person to virtual programming. The majority of groups and patients were not provided the ability to self-select format. All patients discharged between January 1, 2020, and March 17, 2021, were considered eligible participants categorized as those who attended IOP in person and were contacted to participate.

Patient data were collected at baseline (within 30 days of admission) and at 6 post discharge follow-up points. This study uses only baseline and administrative treatment data. Patient demographic and electronic health record (EHR) data related to IOP episode–level information (eg, length of stay, discharge status, and number of sessions attended) were acquired from HBFF’s EHR database management system. One-time baseline surveys were administered to clinicians from December 2020 through March 2021. Clinicians were assessed on demographic characteristics, professional background, and clinical constructs relevant to virtual and in-person IOP including measures of empathy, resistance to change, and comfort with technology. Baseline surveys clinicians were administered by HBFF research staff who were systematically trained to ensure consistent high-quality data gathering that adhered to patient confidentiality standards [26].

### Participants

Of the 126 clinicians who provided IOP services during the study period, 63 (50% response rate) clinicians responded to the clinician survey. Over 96% (n=61) of responding clinicians fully completed the survey, with 2 removed because of missing data. A total of 1844 participants were removed because their respective clinician either did not respond to the clinician survey or had missing data; 284 were removed because they received care in both groups (hybrid treatment), and 4 were deceased prior to discharge. An additional 57 participants had incomplete EHR data, and consequently, their retention outcomes were not usable. Out of the remaining participants, 70 (<5% of the sample) patients were removed because of missing data on covariates other than education. A total of 406 (28.7% of the analytic sample) remaining patients had missing data on education, and therefore education was recoded as a 3-level variable to include those who had missing education data: some college or less, college or more, and missing.

### Analytic Sample

Those who were single and younger had slightly higher rates of removal due to missing data. Otherwise, there were no major differences between participants who were and were not removed due to clinician response or missing data. Due to patient-level missing data, an additional 2 clinicians were removed, and 1 was removed due to identifying a gender outside of male or female (which subsequently removed 5 patients nested within the removed clinician), yielding a final analytic sample of 1410 patients nested in 58 clinicians.

### Ethical Considerations

The study was reviewed and approved by Emory University’s institutional review board (STUDY00001822) and was determined to have met the human research exemption since all data were collected within the context of the HBFF’s standard routine outcome monitoring practices.

### Measures

#### Outcomes

Treatment retention was measured as the percentage of sessions missed, which was calculated by dividing no-shows by the number of scheduled IOP sessions. Successful discharge with staff approval was a dichotomous measure that captured discharged or transferred with staff approval versus all others (against medical advice, at staff request, conditional with staff approval, medical discharge, transfer against medical or staff advice, transfer at staff request, transfer conditional with staff approval). All means and ranges are reported in Table 1.

**Table 1:** Patient- and clinician-level descriptive variables.

Variables			Mean (SD)	Range	n (%)
**Patient-level variables (N=1410)**					
	**Outcomes**				
		Percentage of no-shows	24.3 (23.7)	0-100	
		Discharged or transferred with staff approval			827 (58.7)
	Virtual IOP^a^ (vs in-person)				1018 (72.2)
	“Stepped down” to IOP (vs “stepped in”)				728 (51.6)
	Multiple substance use disorders				512 (36.3)
	**Substance use disorder (primary)**				
		Alcohol			1200 (85.1)
		Cannabis			319 (22.6)
		Opioid			188 (13.3)
		Sedative			158 (11.2)
		Cocaine			130 (9.2)
		Hallucinogen			8 (0.6)
		Other stimulants			172 (12.2)
		Other psychoactive			23 (1.6)
	Study month		10.8 (3.5)	1-18	
	Sex^b^ (male=1)				891 (63.2)
	Unemployed (vs other)				334 (23.7)
	**Educational attainment**				
		Some college or less			453 (32.1)
		College degree or more			554 (39.3)
		Missing			304 (28.6)
	**Marital status**				
		Married			594 (42.1)
		Single			575 (40.8)
		Divorced or widowed			199 (14.1)
		Cohabitation or life partner			42 (3)
	**Race or ethnicity**				
		Non-Hispanic White			1263 (89.6)
		Hispanic			63 (4.5)
		Non-Hispanic another or multiple			85 (6)
	Patient age		40.0 (12.6)	18-81	
**Clinician-level variables (N=58)**					
	Prefer virtual format (vs other)				8 (13.8)
	Patient load		31.1 (18.9)	1-78	
	**Empathy scale**				
		Perspective taking	3.1 (0.5)	2-4	
		Empathic concern	3.2 (0.5)	2-4	
		Personal distress	0.9 (0.6)	0-2.4	
	Technology comfort scale		3.7 (0.7)	1.9-4.9	
	Stress index		14.2 (6.0)	1-30	
	Resistance to change scale		2.6 (0.7)	1.1-3.9	
	Years with license		7.0 (5.9)	0-37	
	Clinician age^b^		3.3 (1.3)	1-6	
	Gender identity (female=1)				36 (62)
	Race or ethnicity (White=1)				54 (93.1)

^a^IOP: intensive outpatient program.

^b^Clinician age is measured categorically (1: 18-25, 2: 26-35, 3: 36-45, 4: 46-55, 5: 56-65, and 6: 65+ years).

#### Clinician-Level Measures

A dichotomous measure was used to assess whether clinicians preferred virtual treatment formats (1=virtual, 0=hybrid or in-person). The number of patients who clinicians served was captured via a count measure based on aggregating patient sample size within each clinician (count of patients served). Empathy was assessed via 3 subscales such as perspective taking (α=.79), empathic concern (α=.71), and personal distress (α=.83) [27-29] from the Interpersonal Reactivity Index (IRI). Positive values for each subscale were indicative of high levels of each facet of empathy. Perspective taking reflects an ability or proclivity to shift perspectives when interacting with other people (eg, “I try to look at everybody’s side of a disagreement before I make a decision”) [28]. Empathic concern captures the degree to which people feel concerned for an observed individual (eg, “I often have tender, concerned feelings for people less fortunate than me”) [28]. Personal distress captures individuals’ feelings of discomfort at witnessing the negative experiences of others (“When I see someone who badly needs help in an emergency, I go to pieces”) [28]. Response options for each IRI item ranged from “0=do not describe me well” to “4=describes me very well,” and subscales were generated by taking the average of 7 items pertaining to each subscale [28]. Comfort with technology was assessed via the TechPH scale (α=.76) [30], which consisted of an average across 8 items (eg, “Using technology makes life easier for me”; “1=strongly disagree” to “5=strongly agree”); positive values capture more comfort. Stress was captured via an index using the Perceived Stress Scale (α=.89), which was generated by summing 10 items (eg, “In the last month, how often have you felt nervous or stressed”; response options: “0=never” to “4=very often”); high values indicate more perceived stress [31]. Resistance to change was measured via the resistance to change scale (α=.88) [32], which was generated by averaging across 17 items (eg, “I like to do the same old things rather than try new and different ones”; response options: “1=strongly disagree” to “5=strongly agree”); higher values capture more resistance to change. Clinician-level covariates also included the number of years clinicians had their counseling license; a categorical measure of age (18-25, 26-35, 36-45, 46-55, 56-65, and 65+ years); gender identity (male and female); and race or ethnicity (White vs other).

### Analytic Strategy

Since the data for this study had a nested structure (ie, patients were nested within clinicians), 2-level multilevel models (MLMs) were used to assess how clinician-level variables (adjusting for patient-level variables) and were related to treatment retention outcomes [33]. MLM accounts for dependence in error terms, which can potentially occur within nested data, by analyzing patients and clinicians as separate levels of data and by including random effects [33]. Two regression equations are estimated simultaneously—a within-clinician equation (ie, patient-level model) and a between-clinician equation (ie, clinician-level model) [33]. Since the discharge or transfer outcome was a binary measure, logistic MLMs were used. In logistic MLMs, the between-clinician parameters reflect average values that are logistic coefficients rather than normal regression coefficients.

Four sets of models were conducted for each outcome. First, null models assessed all unexplained variance at patient and clinician levels. Second, additional models included all patient- and clinician-level covariates, explaining variance at each level. Third, the authors tested whether random slopes were needed for the relationship between VIOP versus in-person and each outcome (ie, an error term for the coefficient). Finally, MLMs stratified according to patient-level VIOP and in-person IOP were conducted, producing 4 additional models (for each outcome) for each subgroup (virtual and in-person). Sensitivity analyses included MLMs that used multiple imputed data for missing data at the patient level, including missing data on education. The main results did not change with the use of multiple imputed data at the patient level. Consequently, authors used listwise deletion for missing data at the patient level and the 3-level education data that included individuals with the missing education level. Finally, supplemental analyses compared VIOP patients with in-person patients across all measures used in the analysis.

## Results

Table 1 presents patient and clinician characteristics. The average percentage of no-shows was 24.3 (SD 23.7), and 58.7% (n=827) of the sample was discharged or transferred with staff approval without conditions. The majority of participants received virtual (n=1018, 72.2%) compared to in-person IOP treatment. More than 1 (n=512, 36.3%) in 3 had more than 1 SUD diagnosis. The majority (n=1200, 85.1%) had alcohol use disorder as their primary diagnosis, followed by cannabis (n=319, 22.6%), opioids (n=188, 13.3%), other stimulants besides cocaine (n=172, 12.2%), and sedative or hypnotics (n=158, 11.2%). Over half (n=728, 51.6%) “stepped down” into IOP from some higher form of care versus “stepping in” from lower forms of care. The majority of clinicians were female (n=36, 62%), White (n=54, 93.1%), had an average of 7 (SD 5.89) years with a license, and carried an average patient caseload of 31 (SD 18.94) individuals. In total, 3% (n=2) of clinicians were between the ages of 18 and 25 years, 31% (n=18) between 26 and 35 years, 21% (n=13) between 36 and 45 years, 24% (n=14) between 46 and 55 years, 17% (n=10) between 56 and 65 years, and 3% (n=2) over the age of 65 years. Of the sample, only 13.8% (n=8) of clinicians endorsed a preference for a virtual format over providing in-person services.

Null models showed that there was a statistically significant variance in percentages of no-shows and discharged or transferred with staff approval across clinicians. Approximately 6.7% and 11% of the variance in the percentage of no-shows and successful discharge with staff approval outcomes were at the clinician level, respectively. Table 2 shows MLM results for treatment retention outcomes for both VIOP and in-person IOP. Relative to in-person, VIOP was negatively associated with the percentage of no-shows (β=–5.71; *P*=.03) and positively associated with discharges with staff approval (odds ratio [OR] 2.38, 95% CI 1.50-3.76). The personal distress subscale was negatively associated with the percentage of no-shows (β=–6.31; *P*=.003). Variance at the clinician level remained significant after accounting for both patient- and clinician-level variables, and the slope for VIOP varied significantly across clinicians.

**Table 2:** Full multivariable hierarchical regression results for treatment retention outcomes.

Variables			Percentage of no-shows						Discharged with staff approval				
			*b*		SE	*P* value		OR^a^ (95% CI)			SE		*P* value
VIOP^b^			–*5.71*^c^		2.66	*.03*		*2.38* (*1.50-3.76*)			0.56		*.001*
**Clinician-level variables**													
Prefer virtual format (vs other)			5.75		3.13	.07		0.75 (0.37-1.50)			0.27		.41
Patient load			0.03		0.05	.55		1.01 (1.00-1.02)			0.006		.21
**Empathy scale**													
	Perspective taking	–2.61		2.44		.28	0.99 (0.57-1.71)			0.28		.96	
	Empathic concern	–2.09		2.21		.34	1.02 (0.62-1.68)			0.26		.93	
	Personal distress	–*6.31*		2.16		*.003*	1.33 (0.82-2.14)			0.32		.24	
Technology comfort scale			–2.14		1.51	.16		1.20 (0.86-1.67)			0.20		.29
Stress index			–0.11		0.21	.61		0.98 (0.94-1.03)			0.02		.38
Resistance to change scale			1.59		1.68	.34		0.95 (0.65-1.39)			0.18		.80
Years with license			–0.09		0.18	.66		1.03 (0.99-1.07)			0.02		.17
Age^d^			–0.30		0.83	.76		0.99 (0.82-1.19)			0.09		.88
Gender (female=1)			–1.63		1.97	.41		1.36 (0.87-2.12)			0.31		.18
White (vs another race or ethnicity)			3.53		3.77	.35		0.88 (0.38-2.04)			0.38		.76
**Variance components**													
	Clinician level	*47.77*				*<.001*							
	Patient level	*472.64*				*<.001*	*0.27* (*0.12-0.60*)					*<.001*	
	VIOP slope	*56.95*				*<.001*							

^a^OR: odds ratio.

^b^VIOP: virtual intensive outpatient program.

^c^Italic formatting indicates statistical significance at *P*<.05.

^d^Clinician age is measured categorically (1: 18-25, 2: 26-35, 3: 36-45, 4: 46-55, 5: 56-65, and 6: 65+ years).

Table S1 in Multimedia Appendix 1 describes any differences in outcomes and patient and clinician measures between VIOP and in-person IOP groups. Consistent with MLM results, individuals in the virtual group had lower percent no-shows (n=221, 21.71% vs n=314, 30.89%; *P*<.001) and a higher percentage of discharge with staff approval (n=622, 61% vs n=535, 52.55%; *P*=.004) compared to in-person group. Relative to the in-person group, patients in the VIOP group had a higher percentage of alcohol use disorder diagnosis (n=879, 86.35% vs n=834, 81.89%; *P*=.04), a lower percentage of cocaine use disorder diagnosis (n=77, 7.56% vs n=138, 13.51%; *P*=.001), and a lower percentage of having multiple SUDs (n=351, 34.48% vs n=418, 41.07%; *P*=.02). In-person patients were slightly younger (38.09 vs 40.70). Patients in VIOP versus in-person tended to have clinicians that had a greater preference for virtual format (n=135, 13.26% vs n=68, 6.63%; *P*<.001), had more years with a license (6.77 vs 5.51, *P*<.001), and were less likely to be White (n=956, 93.91% vs n=984, 96.68%; *P*=.04).

Table 3 results address retention and discharge outcomes for VIOP. Clinician scores on the personal distress subscale were negatively associated with the percentage of no-shows (β=–6.17; *P*=.01). Female clinician gender was positively associated with discharge with staff approval (OR 1.67, 95% CI 1.04-2.63). There was significant variance in both outcomes at the clinician level for VIOP.

**Table 3:** Full multivariable hierarchical regression results for virtual treatment retention outcomes.

Variables			Percent no-shows^a^						Discharge with staff approval^b^				
			*b*		SE		*P* value		OR^c^ (95% CI)		SE		*P* value
**Clinician-level variables (Clinician, N=56; Patient, N=1018)**													
Prefer virtual format (vs other)			5.21		3.45		.13		0.64 (0.31-1.29)		0.23		.21
Patient load			0.05		0.06		.41		1.00 (0.99-1.01)		0.01		.91
**Empathy scale**													
	Perspective taking	–0.60		2.74		.83		0.98 (0.56-1.74)		0.28		.96	
	Empathic concern	–2.35		2.45		.34		1.04 (0.63-1.73)		0.27		.88	
	Personal distress	–*6.17*^d^		2.43		*.01*		1.27 (0.77-2.09)		0.32		.35	
Technology comfort scale			–1.48		1.69		.38		1.15 (0.81-1.62)		0.20		.44
Stress index			0.002		0.24		.99		1.00 (0.95-1.05)		0.02		.91
Resistance to change scale			1.25		1.87		.50		0.87 (0.59-1.28)		0.17		.47
Years with license			0.01		0.19		.95		1.03 (0.99-1.07)		0.02		.16
Age^e^			–1.02		0.95		.28		0.99 (0.81-1.20)		0.10		.90
Gender (female=1)			–0.86		2.24		.70		*1.66* (*1.04-2.63*)		0.39		*.03*
White (vs another race or ethnicity)			4.13		4.09		.31		1.04 (0.44-2.42)		0.45		.94
**Variance components (Clinician, N=56; Patient, N=1018)**													
	Clinician level	*20.28*				*<.001*		*0.22* (*0.08-0.60*)				*<.001*	
	Patient level	*420.73*				*<.001*							

^a^No-shows are count measures and negative binomial regression was used.

^b^Discharged or transferred with staff approval is binary and logistic regression was used.

^c^OR: odds ratio.

^d^Italic formatting indicates statistical significance at *P*<.05.

^e^Clinician age is measured categorically (1: 18-25, 2: 26-35, 3: 36-45, 4: 46-55, 5: 56-65, and 6: 65 years and older).

Table 4 results highlight in-person treatment retention and discharge outcomes. Clinician scores on the perspective taking and personal distress empathy subscales were negatively associated with the percentage of no-shows (β=–9.22; *P*=.03 and β*=*9.44; *P*=.02, respectively). Female clinician gender was negatively associated with the percentage of no-shows (β=–6.43; *P*=.04). Finally, there was a positive association between patient load and successful discharge with staff approval for in-person treatment (OR 1.04, 95% CI 1.02-1.06). There was no significant variance in both outcomes across clinicians for in-person treatment.

**Table 4:** Full multivariable hierarchical regression results for in-person treatment retention outcomes.

Variables			Percent no-shows^a^							Discharge with staff approval^b^				
			*b*		SE		*P* value		OR^c^ (95% CI)			SE		*P* value
**Clinician-level variables (Clinician, N=39; Patient, N=392)**														
Prefer virtual format (vs other)			9.01		5.91		.13		1.65 (0.47-5.81)			1.06		.43
Patient load			–0.02		0.10		.87		*1.04* (*1.02-1.06*)			0.01		*.001*
**Empathy scale**														
	Perspective taking	–*9.22*^d^		4.10		*.02*		0.79 (0.34-1.81)			0.33		.57	
	Empathic concern	0.57		4.21		.89		1.35 (0.57-3.23)			0.60		.498	
	Personal distress	–*9.44*		3.95		*.02*		0.97 (0.42-2.27)			0.42		.95	
Technology comfort scale			–6.21		2.63		.02		1.61 (0.94-2.76)			0.44		.08
Stress index			–0.22		0.33		.52		0.97 (0.91-1.04)			0.03		.42
Resistance to change scale			1.12		2.89		.70		0.97 (0.54-1.74)			0.29		.91
Years with license			–0.79		0.44		.07		1.03 (0.94-1.13)			0.05		.55
Age^e^			1.41		1.30		.28		0.86 (0.66-1.11)			0.11		.24
Gender (female=1)			–*6.43*		3.14		*.04*		1.18 (0.60-2.30)			0.40		.64
White (vs another race/ethnicity)			–4.71		7.93		.55		0.43 (0.09-2.10)			0.35		.30
**Variance components (Clinician, N=39; Patient, N=392)**														
	Patient level	*587.66*				*<.001*		0.10 (0.01-2.51)					>.05	
	Clinician level	0.00				>.05								

^a^No-shows are count measures and negative binomial regression was used.

^b^Discharged or transferred with staff approval is binary and logistic regression was used.

^c^OR: odds ratio.

^d^Italic formatting indicates statistical significance (*P*<.05).

^e^Clinician age is measured categorically (1: 18-25, 2: 26-35, 3: 36-45, 4: 46-55, 5: 56-65, and 6: 65 years and older).

## Discussion

### Principal Findings

This study is the first to investigate the influence of clinician-level characteristics across both virtual and in-person formats with a large sample size of patients with SUDs receiving care through intensive outpatient programming. Participants in the VIOP treatment had lower no-show rates and a greater percentage of discharges with staff approval compared to in-person treatment, building on previous findings indicating the feasibility of VIOP services for SUD [25]. These results are consistent with past reports of higher rates of patient satisfaction, fewer barriers to treatment attendance, and comparable quality associated with virtual services [34-36].

Significant associations between female clinician gender, patient caseload, and the personal distress subscale of the IRI were identified. Female clinician gender was associated with an increased likelihood of discharge with staff approval in VIOP and a lower rate of percent no-shows in the in-person setting. The significant associations among female clinicians, lower rates of no-shows, and discharges with staff approval corroborate previous research that shows female gender clinicians tend to have better patient outcomes relative to their male gender counterparts [3,12].

The personal distress subscale used in this analysis addresses the clinician’s level of comfort when dealing with emergent situations. There has been limited research on how delivery settings may impact clinicians’ abilities to manage their own discomfort when providing interventions that can elicit a brief increase in clinician distress (such as the clinician’s emotional dysregulation during the delivery of trauma interventions). When comparing across all genders, the personal distress portion of the IRI was negatively associated with the percentage of no-shows for both in-person and virtual treatment formats. This finding implies that when clinician personal distress increases, the percentage of no-shows decreases, which is inconsistent with past literature asserting that the levels of personal distress in a clinician may create a lower therapeutic alliance [37]. One hypothesis is that in clinical practice, therapeutic goals and alliance may be kept on a superficial level if a clinician’s distress level rises with the level of patient distress. Resulting avoidance of distress could potentially appeal to, and better retain, patients by not requiring them to deeply investigate emotionally distressing content. Our results show that effective clinicians have a similar impact on outcomes regardless of the delivery setting. This suggests that it may be prudent for clinicians to develop creative ways to use the same treatment strategies in diverse delivery settings. Clinicians need to be prepared should distressing situations arise and not deviate or avoid difficult content due to their fear that virtual interventions may be less effective.

Further research will be necessary to elucidate this potential relationship. A higher patient caseload was associated with a greater likelihood of discharging with staff approval in the in-person setting. An additional analysis evaluating the relationship between years in the field and patient load, which may occur when senior clinicians have larger caseloads, found no significant results, warranting further investigation in future studies.

Few clinician-level characteristics were significantly associated with rates of no-shows and successful discharge. Comfort with technology and preference for virtual format did not reach significance in either setting. This finding is surprising to the authors since provider comfort and satisfaction with virtual care have been a critical determining factor in sustainability, and their reported ability to successfully use telehealth services has been found to be impactful to patient success and outcomes [38,39]. This result suggests that comfort with technology and preference for virtual care may not be necessary for clinicians to deliver effective treatment. Past surveys have identified clinician-level concerns about the use of virtual services because of challenges with work efficiency, reimbursement, regulatory items, privacy, safety, technology limitations, and difficulty establishing rapport [40-43]. Preference for format in our study was not associated with the outcomes evaluated. Additionally, 2 of the 3 subscales of the empathy measure (perspective taking and empathetic concern) did not reach significance, indicating that these factors may be less important in the delivery of group-based SUD IOP services.

### Strengths and Limitations

To the authors’ awareness, this is the largest prospective longitudinal cohort study to assess the impact of clinician-level factors on patient outcomes within in-person and virtual SUD treatment settings. However, several potential limitations should be considered when interpreting the results. Our analyses used data collected during the COVID-19 pandemic, without the ability to compare outcomes prior to the pandemic. Although the sample is representative of the patient population at HBFF, the majority of both the patient and clinician samples were White and male, representing a potential limitation in generalizability to patient populations with higher rates of minorities and marginalized persons. While mechanisms of therapeutic alliance were not directly measured, measures used in our study used ancillary variables that have been shown to have indirect effects on therapeutic alliance and patient outcomes. In this observational study, the authors were unable to ensure that the compared groups were equivalent because of a lack of randomization. As a result, differences in outcomes between groups should be interpreted with caution. This potential limitation is addressed by a secondary analysis that demonstrates limited differences between groups. Future research should focus on broadening the demographic variables in the sample, collecting additional measures of therapeutic alliance, further examining the relationship between the personal distress scale and patient retention, and investigating outcomes outside of the Hazelden Betty Ford treatment facilities to enhance generalizability. Despite these limitations, the findings are an ecologically valid examination of in-person and virtual care within a current health care system providing SUD treatment.

### Conclusions

This study investigated the potential influence of clinician characteristics on patient outcomes through virtual and in-person treatment modalities. Patients in VIOP had lower rates of no-shows and discharges with staff approval. Overall, there were no specific clinician-level characteristics that were positively associated with patient outcomes, including comfort with technology and format preference. Further research is necessary to better understand the identified associations between male clinician gender, patient load, and the relationship between the personal distress subscale and patient retention. These findings help to elucidate the role of clinician characteristics in the effective delivery of SUD treatment, particularly as the field continues to investigate virtual treatment delivery.

## Appendix

Multimedia Appendix 1 Differences in outcomes, as well as patient and clinician measures across treatment delivery settings.

## Abbreviations

DSM-IV: Diagnostic and Statistical Manual of Mental Disorders, Fourth Edition
EHR: electronic health record
IOP: intensive outpatient program
IRI: Interpersonal Reactivity Index
HBFF: Hazelden Betty Ford Foundation
MLM: multilevel model
OR: odds ratio
SUD: substance use disorder
VIOP: virtual intensive outpatient program
