# Supplementary material for: Association Between Clinician-Level Factors and Patient Outcomes in Virtual and In-Person Outpatient Treatment for Substance Use Disorders: Multilevel Analysis
Source: JMIR Hum Factors. 2023 Nov 3;10:e48701. doi: 10.2196/48701 (PMC10656667; doi:10.2196/48701)
Supplement: Supplementary file 1 [file humanfactors_v10i1e48701_app1.docx]

Supplemental Table 1. Differences in Outcomes, and Patient and Clinician Measures across Treatment Delivery Setting

Treatment Delivery Setting:	Virtual		In Person			Total	
Variables	% (N) or Mean (S.D.)				P-value		
Main outcomes							
Percent of no-shows	**21.71%**	**(22.17)**	**30.89%**	**(26.16)**	**<0.001**	24.26%	(23.70)
Discharge with staff approval	**61.00%**	**(621)**	**52.55%**	**(206)**	**0.004**	58.65%	(827)
Patient-level measures							
"Stepped down" to IOP (vs. "stepped in")	52.36%	(533)	49.49%	(194)	0.33	51.56%	(727)
Multiple substance use disorders (vs. only 1)	**34.48%**	**(351)**	**41.07%**	**(161)**	**0.02**	36.31%	(512)
Substance use disorder diganosis							
Alcohol	**86.35%**	**(879)**	**81.89%**	**(321)**	**0.04**	85.11%	(1200)
Cannabis	21.41%	(218)	25.51%	(100)	0.10	22.55%	(318)
Cocaine	**7.56%**	**(77)**	**13.51%**	**(53)**	**0.001**	9.22%	(130)
Hallucinogen	0.49%	(5)	1.02%	(4)	0.26	0.64%	(9)
Opioid	12.28%	(125)	15.82%	(62)	0.08	13.26%	(187)
Sedative	11.39%	(116)	10.71%	(42)	0.72	11.21%	(158)
Other stimulant	11.69%	(119)	13.52%	(53)	0.35	12.20%	(172)
Other psychoactive	1.67%	(17)	1.28%	(5)	0.59	1.56%	(22)
Study month	**12.58**	**(2.22)**	**6.13**	**(1.47)**	**<0.001**	10.78	(3.54)
Sex (male=1)	62.28%	(634)	65.56%	(257)	0.25	63.19%	(891)
Unemployed (vs. other)	22.79%	(232)	26.02%	(102)	0.20	23.69%	(334)
Educational attainment							
Some college or less	33.89%	(345)	27.55%	(108)	0.06	32.13%	(453)
College degree or more	37.82%	(385)	43.11%	(169)	0.06	39.29%	(554)
Missing	28.29%	(288)	29.34%	(115)	0.06	28.58%	(403)
Marital status							
Married	43.42%	(442)	38.78%	(152)	0.82	42.13%	(594)
Single	38.90%	(396)	45.66%	(179)	0.16	40.78%	(575)
Divorced/widowed	14.73%	(150)	12.50%	(49)	0.16	14.11%	(199)
Cohabitation/life partner	2.95%	(30)	3.06%	(12)	0.16	2.98%	(42)
Race/ethnicity							
Non-Hispanic white	89.19%	(908)	90.56%	(355)	0.72	89.57%	(1263)
Hispanic	4.72%	(48)	3.83%	(15)	0.72	4.47%	(63)
Non-Hispanic other	6.09%	(62)	5.61%	(22)	0.72	5.96%	(84)
Age	**40.70**	**(12.50)**	**38.09**	**(12.75)**	**<0.001**	39.98	(12.62)
Clinician measures (across patients)							
Prefer virtual format (vs. other)	**13.26%**	**(135)**	**6.63%**	**(26)**	**<0.001**	11.42%	(161)
Patient load	**41.37**	**(17.94)**	**45.19**	**(16.54)**	**<0.001**	42.43	(17.64)
Empathy scale							
Perspective taking	3.06	(0.52)	3.00	(0.50)	0.05	3.04	(0.51)
Empathic concern	3.24	(0.49)	3.28	(0.40)	0.18	3.25	(0.46)
Personal distress	0.94	(0.58)	0.89	(0.57)	0.18	0.92	(0.58)
Technology comfort scale	3.68	(0.64)	3.75	(0.58)	0.06	3.70	(0.63)
Stress index	14.33	(6.46)	15.04	(6.42)	0.07	14.53	(6.45)
Resistance to change scale	2.68	(0.69)	2.62	(0.61)	0.16	31.09	(0.67)
Years with license	**6.77**	**(5.70)**	**5.51**	**(3.51)**	**<0.001**	6.42	(5.21)
Age	3.19	(1.28)	3.18	(1.24)	0.74	3.19	(1.27)
Gender identity (female=1)	62.18%	(633)	58.42%	(229)	0.19	61.13%	(862)
Race/ethnicity (white=1)	**93.91%**	**(956)**	**96.68%**	**(379)**	**0.04**	94.68%	(1335)

Note. Ns are reported in right-hand column; SUD=substance use disorder; p-values are presented for comparisons between virtual and not virtual within each group--statistical tests were t-tests for continuous measures, χ^2 tests for dichotomous measures, and Wilcoxon rank-sum tests for ordinal measures. Bolded differences were statistically significant ($p<0.05$).
